# Neurofeedback Reduces P300 Amplitudes to Intensely Emotive Pictures in Depressed Cancer Patients

**DOI:** 10.1177/15500594241287961

**Published:** 2024-10-21

**Authors:** Marvin Krawutschke, Martin Teufel, Kira Schmidt, Saskia Pasche, Theresa Schweig, Anna Bialek, Axel Kowalski, Mitra Tewes, Martin Schuler, Dirk Schadendorf, Norbert Scherbaum, Eva-Maria Skoda, Madeleine Fink, Bernhard W. Müller

**Affiliations:** 1 Clinic for Psychosomatic Medicine and Psychotherapy, 27169 LVR-University Hospital Essen, University of Duisburg-Essen, Essen, Germany; 2 West German Cancer Center, 39081 University Hospital Essen, University of Duisburg-Essen, Essen, Germany; 3 Center for Translational Neuro- and Behavioral Sciences (C-TNBS), University of Duisburg-Essen, Essen, Germany; 4 NeuroFit GmbH, Krefeld, Germany; 5 IB University of Applied Health and Social Sciences, Berlin, Germany; 6 Department of Palliative Medicine, West German Cancer Center, 39081 University Hospital Essen, University of Duisburg-Essen, Essen, Germany; 7 West German Cancer Center, Department of Medical Oncology, 39081 University Hospital Essen, University of Duisburg-Essen, Essen, Germany; 8 German Cancer Consortium (DKTK), 39081 Partner Site University Hospital Essen, and German Cancer Research Center (DKFZ), Essen, Germany; 9 Clinic for Dermatology, 39081 University Hospital Essen, Essen, Germany; 10 LVR-University Hospital Essen, Department of Psychiatry and Psychotherapy, Medical Faculty, University of Duisburg-Essen, Essen, Germany; 11 Department of Psychology, 26603 University of Wuppertal, Wuppertal, Germany

**Keywords:** P300, neurofeedback, EEG, depression, cancer patients

## Abstract

*Objective.* Electroencephalographic neurofeedback (EEG NF) or its effects on event-related potentials (ERPs) in quantitative EEG have not yet been systematically studied in cancer patients. The aim of this study was to investigate the emotional arousal and valence effects on the event-related P300 in a visual oddball paradigm by an individualized EEG alpha and theta/beta NF intervention in cancer patients and survivors (*N *= 18, age between 31 and 73 years). *Methods*. ERPs to low and high arousal target stimuli with either emotional positive or negative content and depressive state were obtained in cancer patients before and after a five-week NF intervention in a waitlist paradigm, following the consensus on the reporting and experimental design of clinical and cognitive-behavioral NF studies (CRED-nf checklist). *Results*. Overall, P300 amplitudes decreased significantly (*p *< .05) from pre to post therapy. Effects concerning high arousal stimuli with negative and positive valences were on the border to significance. Moreover, patients achieved significant relief of depressive symptoms (*p *< .05). Especially younger participants (<55 yrs.) benefited. *Conclusions*. P300 observations could reflect a therapeutic effect on brain activity level. EEG NF alleviates depressive symptoms in cancer patients. *Significance*. Based on these findings, further studies are needed to investigate the effects on event-related potentials by NF therapy.

## Introduction

Event-related potentials (ERPs) are used to explore the neural basis of cognition. One of the more extensively studied ERP components is the P300 (or P3), which was first described in 1965 as a positive potential elicited around 300 ms after stimulus occurrence.^1^ The P300 can be elicited from an oddball task, in which subjects have to respond to a target stimulus presented in-between recurrent standard stimuli. In a visual oddball paradigm, ERPs can be calculated for rare emotionally significant pictures. Today, the P300 is understood as a marker to represent an information processing cascade of attention and memory mechanisms, particularly in the context of stimulus discrimination.^2^

Taking into account processes of contextual updating, the main influencing factors of P300 amplitude were described as task relevance, motivational importance, and arousal level.^3^ Research suggests that the emotional arousal dimension affects the P300 in the sense that subjects who react with high arousal generate larger amplitudes in contrast to people who react with lower arousal.^4,5^

Valence and arousal dimensions of emotion can differentially affect the P300.^6^ More precisely, valence effects were found to be reflected by amplitude modulations at frontal sites.^7^ Arousal effects were observed at posterior locations for several EEG components and in particular for the P300.^8–11^ The arousal effect was more reproducible than the valence effect and seems to be the primary determinant of affective oddball processing.^12^

Various studies investigated ERPs in depressive patients, many of which have shown reductions in P300 amplitudes^13–15^ and increases in P300 latency.^14,16,17^ For example, Brush et al (2022) showed that decreased Go and No-Go P300 amplitudes were associated with increased depressive symptom severity in older adults, with the greatest variance attributable to decreased No-Go P300 amplitude. Of note, loneliness significantly moderated the relationship between No-Go P300 and depressive symptom severity in that individuals who reported low levels of loneliness had no association between No-Go P300 and depressive symptom severity. This suggests that social support provides protection against the depressive effects of poor inhibitory control in older adults.^18^ An increase of the amplitude in the sense of a normalization after recovery from depression was reported only sporadically.^19,20^ Since previous literature suggests a significant reduction in P300 amplitudes in depressive patients, successful therapeutic treatment might result in increased P300 amplitudes.

Cancer patients and survivors suffer from various physical and psychological symptoms like pain, fatigue, anxiety, depression, and cognitive impairments. Depression is the most common and severe comorbidity of cancer patients^21^ with a prevalence of 15% with regard to major and 20% to minor depression.^22^ Overall, it is known that especially older people are affected by depression.^23^ Cancer being the second leading cause of death worldwide with an estimated 9.9 million deaths in 2020^24^ underscores the importance of this research topic.

The technique of electroencephalographic (EEG) biofeedback or neurofeedback (NF) is a non-invasive, non-drug type of brain training and was developed in the 1970s.^25^ It measures the EEG signal of a person, processes it in real time, and extracts different parameters to be trained, mostly by analyzing the frequency content of the signal.^26^ The information is therefore presented in visual or auditory form. The aim of NF therapy is to modify behavior by modeling brain activity through “voluntary control”.^25^ The technique is scientifically based and belongs to the innovative complementary and alternative medical therapies.^27^ By modifying brain waves and thus leading to clinical changes in disease-related symptoms, NF enables the brain to learn self-regulation skills.^28^ Changes in the amplitude of selected frequency bands through operant and classical conditioning lead to a training effect and thus to symptom relief.^27^

Neurophysiological targets can be generally classified into three categories according to general mechanisms^25^: arousal,^29^ emotional valence,^30^ and sleep.^31^ With respect to the main frequency bands, the literature predominantly assigns the posterior alpha rhythm to a state of relaxed wakefulness^32^ and the central frontal theta and beta activity to a state of arousal.^33,34^ Therefore, an increase of alpha frequency by alpha-NF training is applied to enhance the relaxation state and a decrease of the theta/beta frequency is attempted to decrease the arousal state.

So far, NF has been studied in patients with attention/hyperactivity disorder, addiction disorders, strokes, epilepsies, migraines, chronic insomnia, autism spectrum disorder, depression, and psychotic disorders.^25^ Some studies and a recent review suggest that NF has the potential to relieve several long-term symptoms reported by cancer patients and survivors and to improve their quality of life.^35,36^

Hetkamp et al postulated^26^ the need to conduct studies with pre and post EEG data after NF training or therapy “*as standardized outcome measurement to answer the question whether cancer related anxiety and depression are modulated in the same way as the psychiatric findings implicate”*. To date, this has only been investigated in a few case studies within this patient group (eg^37^).

On this basis, the aim of this study was to determine the emotional arousal and valence effects on event-related P300 by an individualized EEG alpha and theta/beta NF intervention in cancer patients and survivors. We expected that a division of the emotional stimuli into high versus low arousal will lead to an increase of the amplitudes of these two ERPs. In the sense of normalization, this increase of the arousal-dependent amplitude will increase during and after successful NF training.

## Methods

### Study Design

The current study is part of a larger randomized, controlled clinical trial registered at the German Clinical Trials Register (ID: DRKS00015773). The study was approved by the ethics committee of the medical department of the University of Duisburg-Essen (No.: 18-8079-BO).

The study was designed as a waiting-list control study. First, EEG (t0) measurement was performed after written informed consent was given. After a five-week waiting period, another EEG (pre, t1) was recorded before patients received the NF intervention, which lasted five weeks as well. Following the intervention, a post EEG (t2) was conducted. In addition, on the same days as the EEG measurements were performed, patients were asked to answer questionnaires.

Regarding EEG data acquisition and analyses, the study was designed single-blinded. In conducting and reporting this clinical trial, we followed the *consensus on the reporting and experimental design of clinical and cognitive-behavioral neurofeedback studies* (CRED-nf checklist).^38^

### Participants

The study was conducted from October 2018 to December 2020. It was interrupted during the first COVID-19 pandemic shutdown phase in Germany from March 23, 2020 to May 6, 2020.

Twenty-one cancer patients or survivors were included in the study. Both patients currently undergoing cancer therapy as well as patients in remission participated. Three patients dropped out due to the COVID-19 pandemic. A sample of 18 (13 female, 5 male) Caucasian patients with a mean age of 53.06 years (min = 31, max = 73; median = 54.50; SD = 10.973 years) were included in the evaluation. Seventeen participants were right-handed and one was left-handed. All of them had normal or corrected-to-normal vision and provided written informed consent before the start of study participation. Prior to the experiment, the absence of exclusion criteria was examined (no severe depressive state, no history of neurological disease including neurological metastasis, or drug abuse) with the ICD-10-Checklist^39^ and *Psycho-Onkologische Basisdokumentation.*^40^ Patients had various cancer entities with a median WHO stage of III. Slightly more than half of the patients were still working; Details are given in Table 1.

**Table 1.** Demographics.

		n	Percentage (%)
Gender			
	female	13	72.2
	male	5	28.8
Relationship			
	with partner	14	77.8
	without partner	4	22.2
Employment status			
	employed	10	55.6
	unemployed	0	0
	retired / incapacitated	3	16.7
	on sick leave	4	22.2
	other	1	5.6
Education			
	high school diploma	10	56.6
	secondary school degree („*Realschule*“)	4	22.2
	secondary school degree („*Hauptschule*“)	2	11.1
	no data	2	11,1
Cancer Type			
	breast	6	33.3
	melanoma	3	16.7
	lung	2	11.1
	lymphoma	2	11.1
	pancreas	2	11.1
	angiosarcoma	1	5.6
	endometrial	1	5.6
	multiple myeloma	1	5.6
Cancer Stage			
	I	3	16.7
	II	0	0
	III	7	38.9
	IV	8	44.4

*N *= 18.

Any participant who completed six or more NF sessions was considered a completer. All participants who completed the study met this condition, the median session number was nine (range 6–10).

### EEG Acquisition

EEG was recorded using a 32-channel Brain Products Inc. (Gilching, Germany) DC EEG amplifier and matching hoods with active, sintered Ag-AgCl electrodes (Acti-Cap slim). Brain Products software (Brain Products Recorder, Version 2) was used for EEG data acquisition. EEG was recorded at a sampling rate of 1000 Hz in the frequency range from 0.016 to 250 Hz across all 32 channels according to the extended 10–20 system: Fp1, Fp2, F7, F3, Fz, F4, F8, Fc7, Fc3, Fcz, Fc4, Fc8, T3, C3, Cz, C4, T4, Tp5, Cp3, Cpz, Cp4, Tp6, T5, P3, Pz, P4, T6, Oz, and left and right earlobes. To control for eye movements, horizontal EOG was recorded from two electrodes at the left and right outer canthi of both eyes. Vertical EOG was recorded from below the left eye and electrode Fp2. Electrode Fz served as reference, the ground electrode was mounted between Fz and Fpz. Visual stimuli and patients’ responses were managed using Presentation software (Version 20.2, Neurobehavioral Systems, Inc., CA, USA). Stimulus events and responses were registered by the EEG recording software.

#### Oddball Task

Pictures from the International Affective Picture System (IAPS) were used for stimulation.^41^ 560 standard stimuli (75,0%) and 35 deviants for each emotion condition (6,25%) were presented. Deviants were positive and negative valence and high and low arousal. This adds to a total of 700 stimuli. With 2.5 s SOA, the presentation lasted about 30 min. All neutral pictures were non-target trials. The presentation of these neutral pictures was randomly interspersed with pictures with either low or high arousal and with either positive or negative valence, resulting in four classes of target stimuli: low-positive (5%), high-positive (5%), low-negative (5%), and high-negative (5%). Participants were instructed to press a button on these emotionally significant pictures.

The oddball task comprised a total of 230 emotional target pictures. The mean (SD) arousal and valence ratings, respectively, were as follows: low-arousal-negative-valence = 4.512 (0.345) and 2.86 (0.31), high-arousal-negative-valence = 6.483 (0.321) and 2.87 (0.45); low-arousal-positive-valence = 4.44 (0.251) and 7.232 (.223); high-arousal-positive-valence = 6.547 (0.399) and 7158 (.372). IAPS picture sets differed significantly in the valence dimension (on a scale ranging from 1 - very unpleasant to 9 - very pleasant; *F*(4,12) = 13454.8, *p *≤ .05). In the arousal dimension (scale ranging from 1 - very relaxing to 9 - very exciting), the unpleasant set and the pleasant set both differed from the neutral one (*F*(4,3) = 41242.9, *p *≤ .05).

#### EEG Procedure

Participants sat with attached electrodes on a comfortable chair in a secluded and dimly lighted room, which was minimized for electrical artifacts. The chair stood at a distance of 1.5 meters from the presentation monitor, on which the picture stimuli were presented. Patients were asked to judge the valence of the randomly presented pictures by pressing two different keys on a computer mouse (randomized for left and right) for the pleasantness of the pictures (positive vs negative); no tap for neutral pictures in their assessment. All participants were instructed to avoid blinking and to maintain their gaze on the black centered cross, which appeared on the computer screen between stimulus presentations.

#### EEG Data Analyses

In a first step, EEG data was controlled for gross artifacts resulting, for example, from subject, jaw, or complex eye-movements. These were excluded from further analysis. Data was then re-referenced to left and right earlobes as new reference followed by the application of a bandpass finite impulse response filter between 0.032 and 30 Hz. Eye blinks and eye-movements were corrected using independent component analysis as implemented in the Analyzer Software. Following stimulus category specific segmentation (−100 ms to 1000 ms) and baseline-correction of segments (−100-0 ms), data was averaged for each individual and each stimulus condition:^42^ standard (neutral), low- and high-arousal-positive valence, and low- and high-arousal-negative-valence stimuli. From the single-subject averages, positive peak amplitudes of the P300 component at electrode Pz were identified in the time-range of 250 ms to 800 ms following stimulus onset and exported for further statistical analyses.^43–45^

### Depression Measurement

The German version of the Patient Health Questionnaire PHQ-9^46^ is a 9-item depression scale. A score under 5 is used as an indicator for the absence of a depressive disorder. Scores ranging from 5 to 9 can mostly be found in patients with a slight or subliminal depressive disorder that can be interpreted as a mild severity. Patients with a major depression most likely score higher than 9 with a moderate (10–14), strong (15–19), or highest (20–27) level of severity.

### NF Intervention

**Figure 1.**
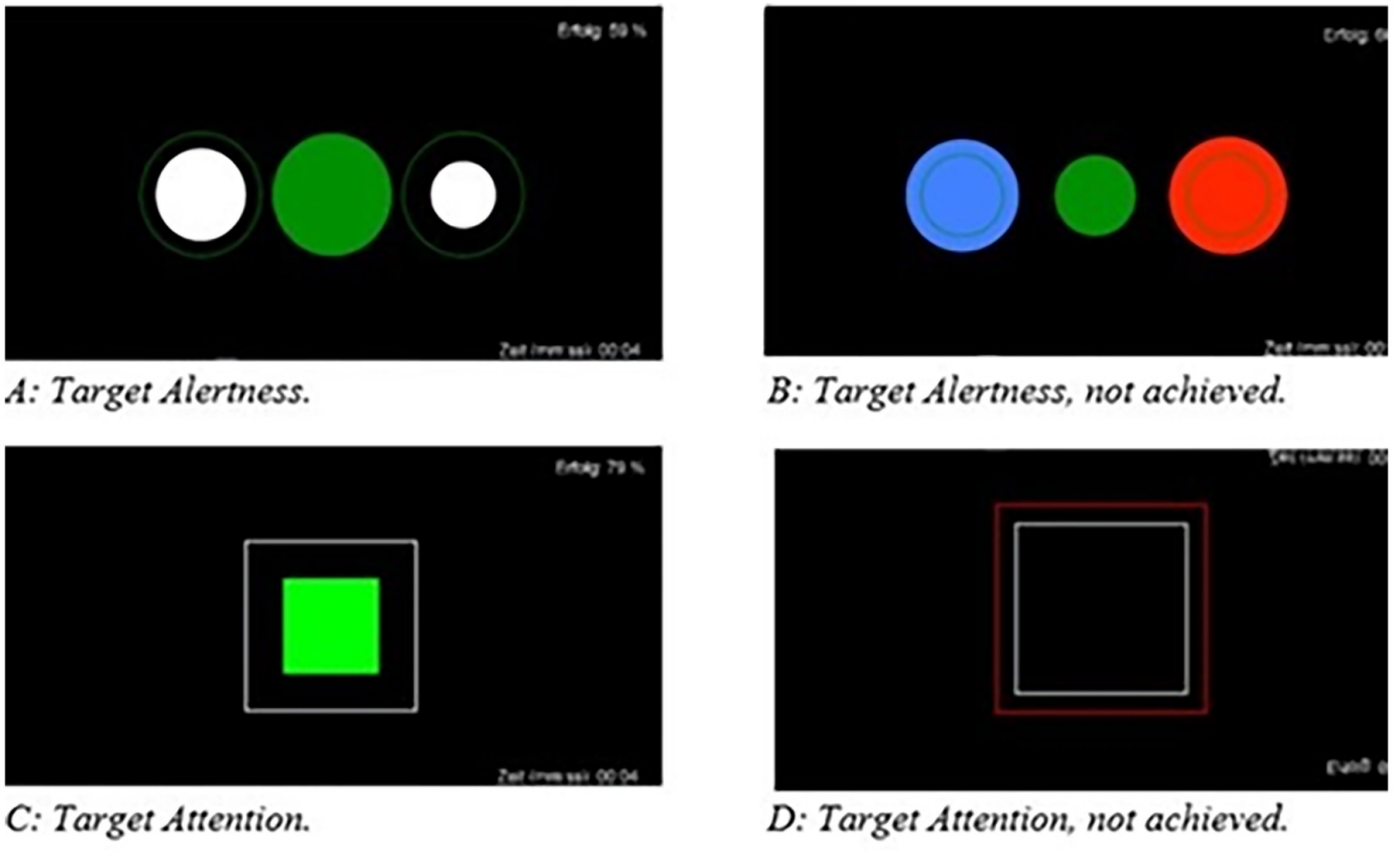
Visual feedback as displayed to participants: A: achieving target alertness; B: target alertness, not achieved; C: achieving target attention; D: target attention, not achieved.

NF training was performed by using the *Mind Wave Headset* (NeuroSky & Inc, 2011) with the electrode being placed on Cz. *BioEra Pro* software (PROATECH LLC) was used to transfer signals from the headset to the computer for further processing. The raw signals were decomposed into individual frequency bands in real time within the software using Fast Fourier Transformation. The output signal was then used for feedback. The stimuli were simple geometric figures (Figure 1) programmed in *Processing*. The clinical examiner (MF) was present during the entire NF training but did not give any verbal feedback during the sessions. The feedback was displayed on the monitor as a target state during the training. Each intervention included a minimum of 6 and a maximum of 10 training sessions over 5 weeks, which resulted in practicing twice a week. Each session consisted of a 35 to 40 min paradigm:

- *Resting-State* for about 5 min
- Alpha-Training (9-13 Hz moderation) and reduction of Theta/Beta (> 20 Hz) for 10 min
- *Resting-State* for about 5 min
- Target: Theta/Beta-Ratio ≤ 2,5 for about 5 min
- *Resting-State* for about 5 min
- Alpha-Training (9-13 Hz moderation) and reduction of Theta/Beta (> 20 Hz) for 10 min

### Statistical Analyses

The statistical evaluation of the specific parameters was conducted with the program *Statistical Program for Social Sciences SPSS* (Version 26.0, New York: IBM Corp.). The level of significance for all analyses was set at α = .05. Statistical outliers with a value higher or lower than twice the standard deviation (± 2SD) for the main outcome variable ΔPHQ(t2-t1) were identified via boxplot and excluded from further analysis (n = 1). Repeated measurement analysis of variance (MANOVA, t0, t1, t2) and age as covariate was conducted to show the depression effects over time. Regarding the P300 effects, the focus remained on the intervention effect (factor therapy), so explicitly only the measurement time points before and after the NF intervention were included in the analyses. For these effects, a general linear model was conducted. For analyses of age effects, a median split (< 55 and > 55 years of age) was performed. Descriptive statistics are shown in Table 2.

**Table 2.** Descriptive Statistics.

Outcome	*N*	*M*	*SD*	*S (SE)*	*K (SE)*
Depressive symptoms					
PHQ t0	18	8.88	4.581	.634 (.536)	−.030 (1.038)
PHQ t1	18	7.71	4.254	.363 (.536)	−.185 (1.038)
PHQ t2	18	6.71	4.165	.165 (.536)	−.912 (1.038)
ΔPHQ(t1-t0)	18	−1.17	2.721	−.531 (.536)	1.523 (1.038)
ΔPHQ(t2-t0)	18	−2,18	3.107	.083 (.536)	.498 (1.038)
ΔPHQ(t2-t1)	17*	−1.00	1.904	−.739 (.550)	.071 (1.063)
Age effects in depressive symptoms					
Age	18	53.06	10.973	−.652 (.536)	.387 (1.038)
PHQ t0 Patients <55 yrs.	9	9.22	4.116	.446 (.717)	−1.631 (1.400)
PHQ t1 Patients <55 yrs.	9	7.78	3.768	.023 (.717)	−1.536 (1.400)
PHQ t2 Patients <55 yrs.	9	6.11	3.621	.108 (.717)	−1.207 (1.400)
PHQ t0 Patients ≥55 yrs.	7**	7.00	3.464	−.303 (.794)	−.461 (1.587)
PHQ t1 Patients ≥55 yrs.	7**	6.29	3.546	−.517 (.794)	−1.762 (1.587)
PHQ t2 Patients ≥55 yrs.	7**	6.29	4.071	−413 (.794)	−2.471 (1.587)
ΔPHQ(t1-t0) Patients <55 yrs.	9	−1.44	3.358	−471 (7.717)	.996 (1.400)
ΔPHQ(t2-t1) Patients <55 yrs.	9	−1.67	2.291	−045 (7.717)	−.885 (1.400)
ΔPHQ(t1-t0) Patients ≥55 yrs.	7**	−.71	2.059	.108 (.794)	−2.051 (1.587)
ΔPHQ(t2-t1) Patients ≥55 yrs.	7**	.00	.816	.000 (.794)	−1.200 (1.587)
P300 amplitude					
P300 poslow t0	18	10.797	3.855	−.090 (.536)	.278 (1.038)
P300 poshigh t0	18	11.206	4.570	.460 (.536)	−039 (1.038)
P300 neglow t0	18	10.766	5.069	.760 (.536)	.691 (1.038)
P300 neghigh t0	18	11.090	4.770	.357 (.536)	−1.066 (1.038)
P300 poslow t1	18	11.972	5.955	−.644 (.536)	−686 (1.038)
P300 poshigh t1	18	12.000	6.161	−.411 (.536)	−1.181 (1.038)
P300 neglow t1	18	11.149	6.959	−.001 (.536)	−.700 (1.038)
P300 neghigh t1	18	12.512	7.349	.026 (.536)	−1.315 (1.038)
P300 poslow t2	18	10.485	5.708	.975 (.536)	.289 (1.038)
P300 poshigh t2	18	11.193	6.199	−.032 (.536)	−.765 (1.038)
P300 neglow t2	18	9.420	5.740	.820 (.536)	1.051 (1.038)
P300 neghigh t2	18	11.144	5.590	.983 (.536)	1.278 (1.038)
ΔP300 poslow (t2-t1)	18	−1.487	5.417	−.967 (.536)	.627 (1.038)
ΔP300 poshigh (t2-t1)	18	−.808	4.417	−1.712 (.536)	2.933 (1.038)
ΔP300 neglow (t2-t1)	18	−1.729	6.771	−.791 (.536)	.652 (1.038)
ΔP300 neghigh (t2-t1)	18	−1.3686	6.158	−1.119 (.536)	.730 (1.038)

* Statistical outliers SD ≥ 2; n = 1.

** Statistical outliers SD ≥ 2; n = 2.

Abbreviations: PHQ = Patient Health Questionnaire PHQ-9,^46^ ΔPHQ(t1-t0) = difference between the mean PHQ at t1 minus t2, ΔPHQ(t2-t1) = difference between the mean PHQ at t2 minus t1 ; poslow = positive valence low arousal; poshigh = positive valence high arousal; neglow = negative valence low arousal; neghigh = negative valence high arousal; ΔP300 (t2-t1) = difference between the mean PHQ at t2 minus t1.

We computed a repeated measurement ANOVA on electrode Pz, P300 amplitudes with therapy (pre/post), arousal level (low/high), and emotion valence (positive/negative) as within factors and age as covariate. Results are reported with regard to Greenhouse-Geisser correction. While our sample covered a broad age range of 31 to 73 years we used age as a covariate in all relevant analyses. In the following age analyses, two age categories were used by the median split method.

## Results

### P300 Amplitudes

**Figure 2.**
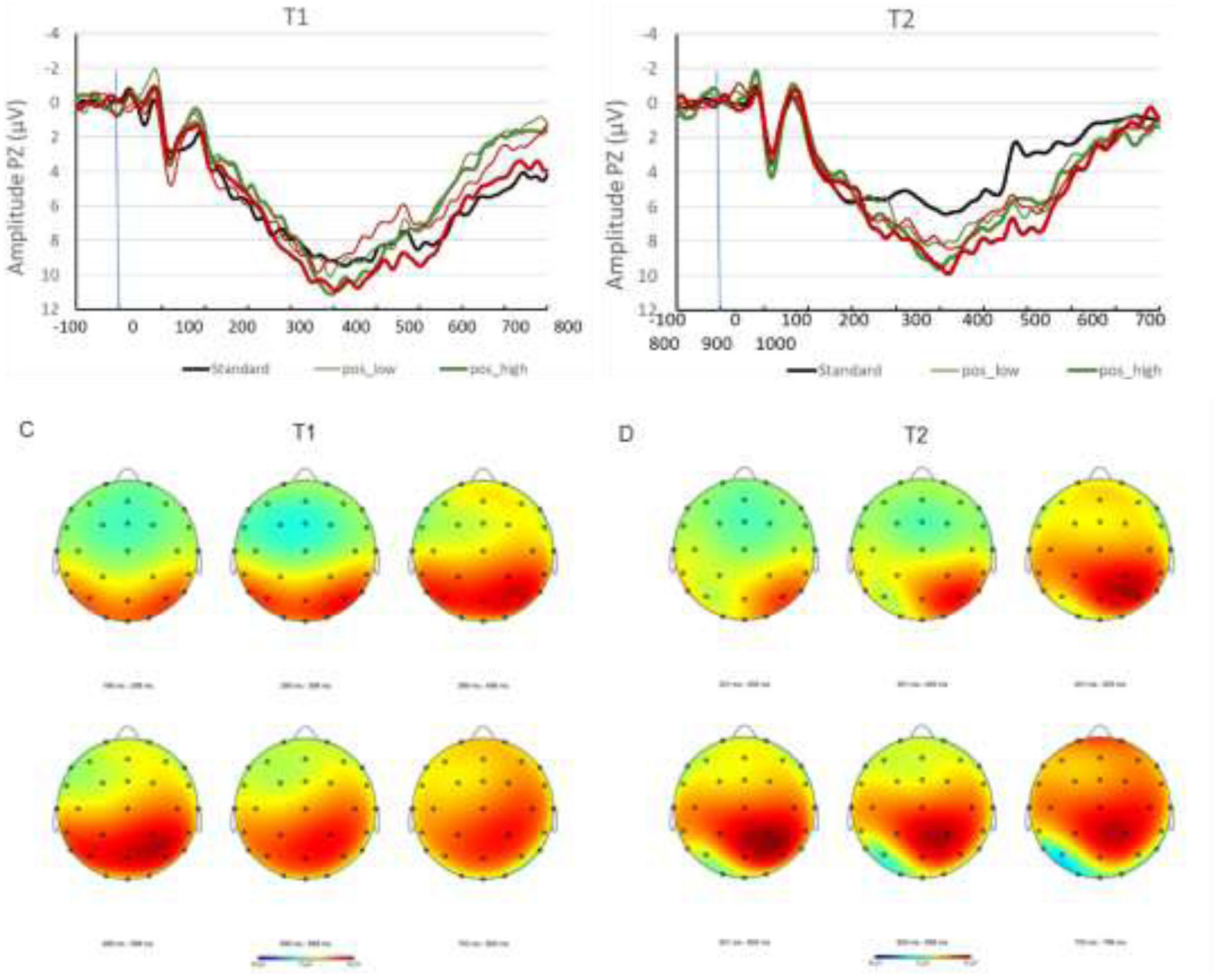
A: Grand averages t1. B: Grand averages t2. C: Headmap t1 D: Headmap t2. Mapping view of evoked potentials across the time-range of the P300 component from 200 ms to 800 ms post stimulus onset, for pre- and post-treatment measurements. Data are collapsed across all four deviant conditions: low and high arousal positive and negative valence stimuli. Amplitude color coding adjusted to a range of −8 µV (blue) to +8 µV (red).

**Figure 3.**
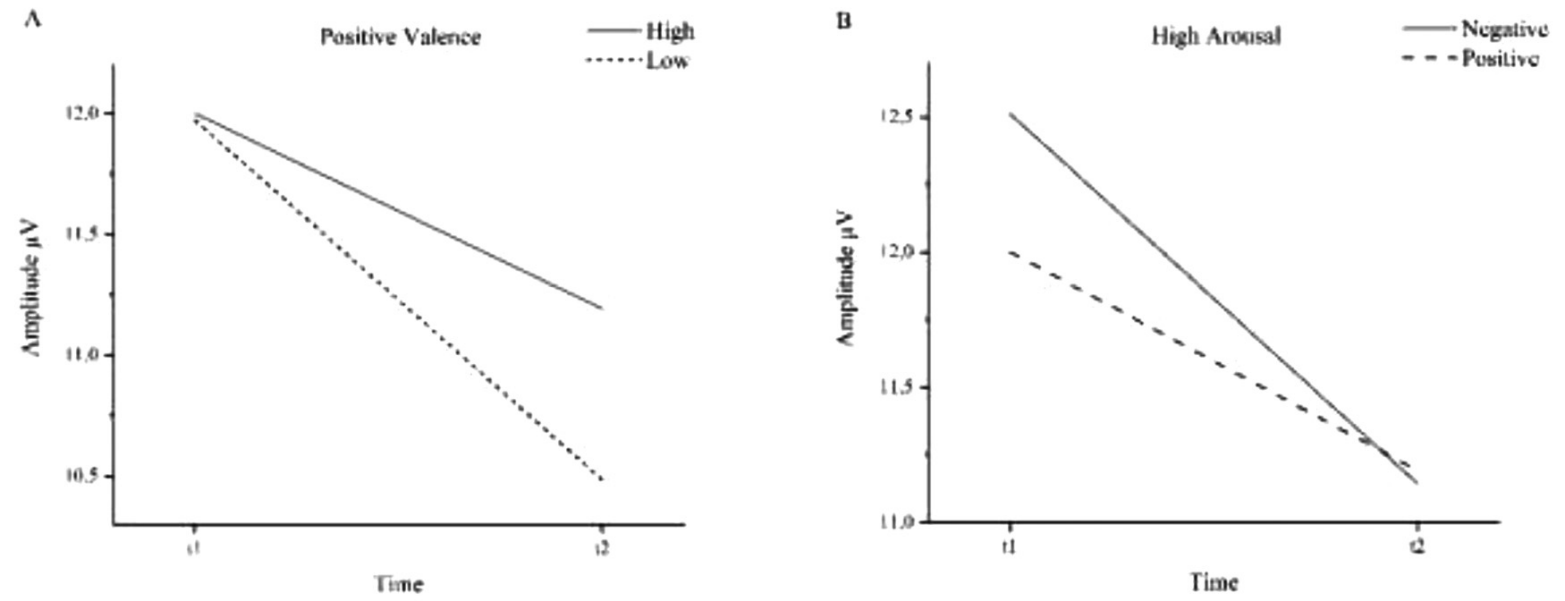
Mean P300 amplitudes before (t1) and after (t2) NF intervention. A: Positive valence stimuli. B: High arousal stimuli.

Figure 2 shows the averaged amplitudes calculated for all 18 patients as grand averages at time point t1 and t2 for each condition. Mean P300 amplitudes before (t1) and after (t2) NF intervention are shown in Figure 3.

**Figure 4.**
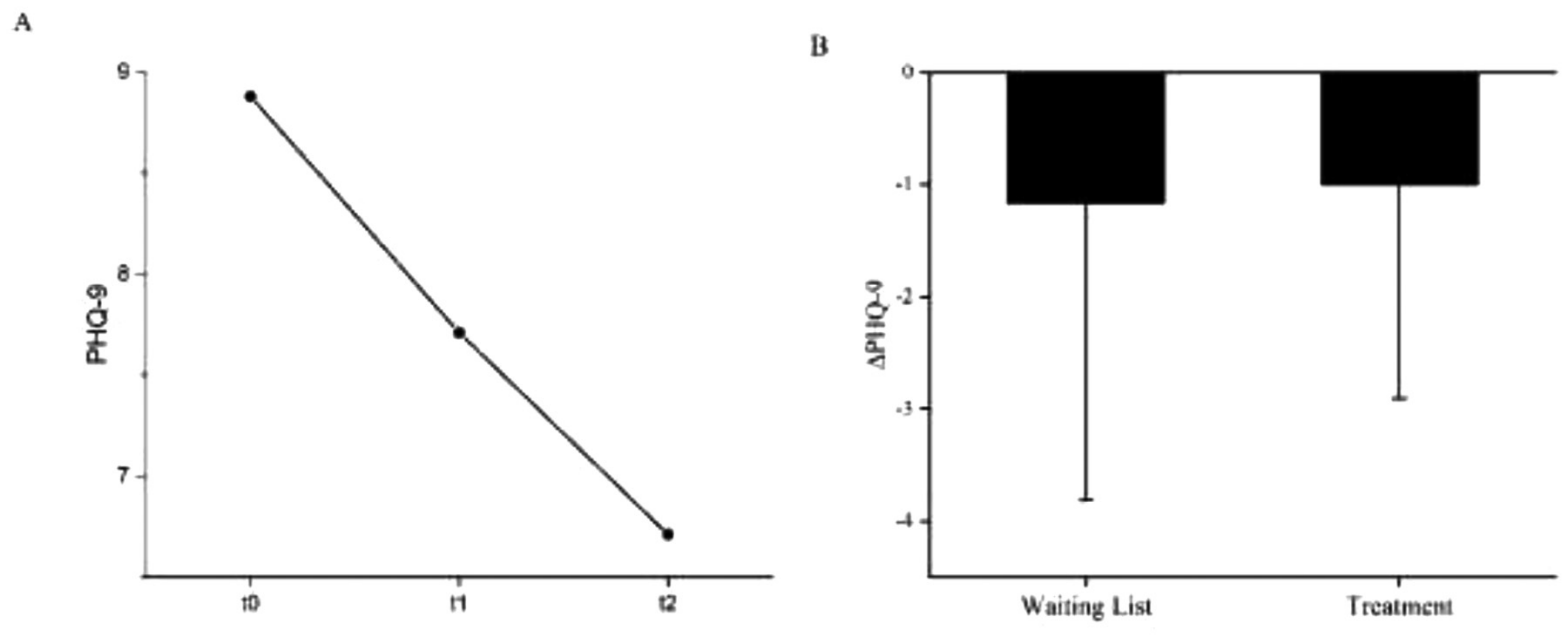
A: Mean values of the depression state (PHQ-9) before waiting period (t0), before (t1) and after (t2) NF treatment. M_PHQ-9 t0 _= 8.88 (4.581), M_PHQ-9 t1 _= 7.71 (4.254), M_PHQ-9 t2 _= 6.71 (4.165). B: PHQ difference between waiting list and NF treatment. ΔPHQ (waiting list) −1.17 (2.640), N = 18; ΔPHQ (treatment) −1.00 (1.904) N = 17*. Error bars show the standard errors (SE). * statistical outliers SD ≥ 2: N = 1.

**Figure 5.**
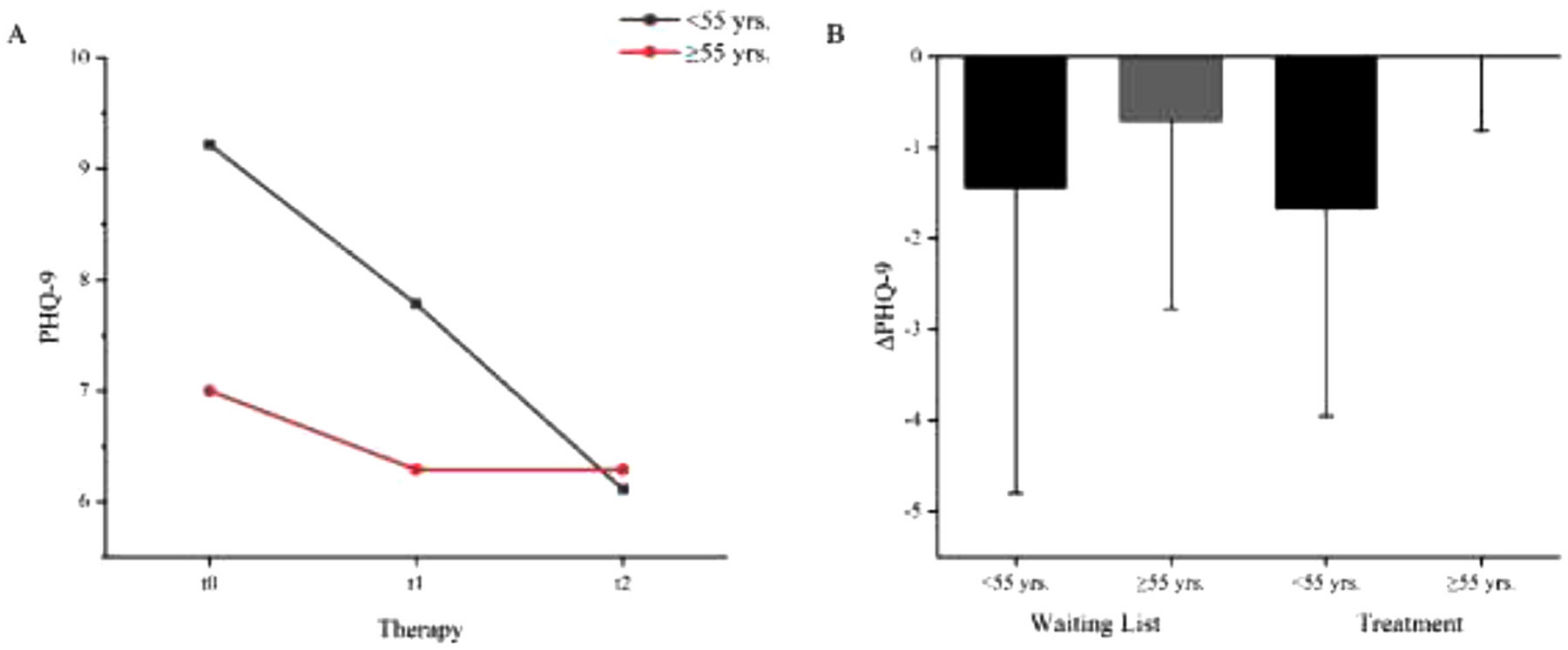
A: Age dependent mean values of the depression state (PHQ-9) before waiting period (t0), before (t1) and after (t2) NF intervention. Patients <55 yrs.: M_PHQ-9 t0 _= 9.22 (4.116), M_PHQ-9 t1 _= 7.78 (3.768), M_PHQ-9 t2 _= 6.11 (3.621), N = 9. Patients ≥55 yrs.: M_PHQ-9 t0 _= 7.00 (3.464), M_PHQ-9 t1 _= 6.29 (3.546), M_PHQ-9 t2 _= 6.29 (4.071); N = 7*. B: Age dependent PHQ difference between waiting list and NF treatment. ΔPHQ (waiting list) < 55 yrs. = −1.44 (3.358), ≥ 55yrs. = −.71 (2.059). ΔPHQ (treatment) < 55yrs. = −1.67 (2.291), ≥ 55yrs. = .00 (0.816). Error bars show the standard errors (SE). * statistical outliers SD ≥2: N = 2.

Overall, P300 amplitudes decreased from pre to post therapy from 11.91 µV to 10.56 µV revealing a significant effect (*F*(1,16) = 12.47, *p *< .05, *η^2^* = .483). Considering age as a covariate, a significant interaction (*F*(1,16) = 11.07, *p *< .05, *η^2^* = .409) with therapy was found.

Other interactions between the main factors therapy, arousal, and valence, or age as the covariate revealed no significant effects.

However, the three-way interaction between therapy, arousal, and valence (*F*(1,16) = 3.97, *p *= .064, *η^2^* = .199) and the interaction between age, therapy, arousal, and valence (*F*(1,16) = 3.846, *p *= .068, *η^2^* = .194) showed trends.

Cronbachs's Alpha revealed different degrees of internal consistency for neutral (*α* = .814), low arousal positive valence (*α* = .725), high arousal positive valence (*α* = .854), low arousal negative valence (*α* = .603), and high arousal negative valence (*α* = .713) stimuli.

Pearson correlation coefficient showed a significant relationship between t1 and t2 measurement for neutral (*r*(16) = .692, *p *= < .001), low arousal positive valence (*r*(16) = .569, *p *= < .05), high arousal positive valence (*r*(16) = .745, *p *= < .001), and high arousal negative valence (*r*(16) = .576, *p *= < .05) stimuli. In the case of low arousal negative valence (*r*(16) = .445, *p *= .064), there was no significant relationship.

### Effects on the Arousal/Valence by Treatment

In order to further understand the complex interaction trend of time, arousal and valence, we computed separate follow-up analyses with time and valence and time and arousal as within factors and age as the covariate (Table 4).

The amplitudes evoked by positive valences decreased significantly from 11.99 µV before therapy to 10.84 µV after therapy (*F*(1,16) = 15.02, *p *= .001, *η^2^* = .484). The interaction between arousal level of positive valence stimuli and therapy showed a positive trend (*F*(1,16) = 3.72, *p *= .072, *η^2^* = .189). Low arousal positive valence stimuli decreased more (11.97 µV to 10.49 µV) than the high arousal positive valence stimuli (12.00 µV to 11.19 µV).

High arousal amplitudes decreased significantly from t1 (12.26 µV) to t2 (11.17 µV; *F*(1,16) = 9.38, *p *< .05, *η^2^* = .370). The interaction between time and valence dimension in the high arousal category showed a positive trend (*F*(1,16) = 4.32, *p *= .054, *η^2^* = .213). Before the intervention, negative valence stimuli (12.51 µV) elicited higher amplitudes than positive valence stimuli (12.00 µV). After the intervention, both amplitudes decreased to a similar level (negative = 11.14 µV, positive = 11.19 µV).

Interactions of therapy and arousal level of negative valence stimuli or valence dimension of low arousal stimuli did not show significant results with or without age as the covariate.

A significant therapy effect of time was observed in each condition considered individually. The interaction between age and therapy on single condition level also revealed significant effects.

### Reaction Time

Mean reaction times between stimulus onset and assessment of valence decreased from t0 to t1 and t2 across all arousal and valence levels with the limitation of large standard deviations. Compared to the other categories, the response time for the strongly negative stimuli was markedly longer. Table 3 show the reaction time in ms.

**Table 3.** Reaction Time in ms.

	*N*	*Min*	*Max*	*Mean*	*SD*
poslow t0	19	249,65	885,78	684,738	214,307
poshigh t0	19	250,91	888,10	681,274	213,841
neglow t0	19	257,99	886,98	679,947	210,693
neghigh t0	19	250,42	2895,57	842,875	536,409
poslow t1	18	209,87	885,30	626,515	238,638
poshigh t1	18	216,36	885,97	627,803	238,071
neglow t1	18	210,95	888,56	627,076	239,329
neghigh t1	18	113,78	11143,93	1215,401	2503,896
poslow t2	17	247,89	887,11	506,968	223,005
poshigh t2	17	245,72	884,47	504,844	222,404
neglow t2	17	247,11	882,07	509,193	221,003

Abbreviations: poslow = positive valence low arousal; poshigh = positive valence high arousal; neglow = negative valence low arousal; neghigh = negative valence high arousal.

**Table 4.** P300 analysis.

Factors	*N*	*F(*1,16)	*p*	*η^2^*
Time	18	12.47	<.05	.483
Arousal	18	1.06	.318	.062
Valence	18	2.12	.164	.117
Time × Arousal	18	2.15	.162	.119
Time × Valence	18	1.10	.310	.064
Time × Arousal × Valence	18	3.97	.064	.199
Age × Time	18	11.07	<.05	.409
Age × Arousal	18	.138	.715	.009
Age × Valence	18	2.73	.118	.146
Age × Time × Valence	18	.92	.352	.054
Age × Time × Arousal	18	1.76	.203	.099
Age × Time × Arousal × Valence	18	3.846	.068	.194
Time × Positive Valences	18	15.02	<.05	.484
Time × Positive Valences × Arousal	18	3.72	.072	.189
Time × Negative Valence	18	9.50	<.05	.373
Time × Negative Valence × Arousal	18	.00	.984	.000
Time × High Arousal	18	9.38	<.05	.370
Time × High Arousal × Valence	18	4.32	.054	.213
Time × Low Arousal × Valence	18	.02	.889	.001
Age × Time × Negative Valence × Arousal	18	.01	.911	.001
Age × Time × Low Arousal × Valence	18	.04	.845	.002
Age × Time × Positive Valence × Arousal	18	1.00	.87	.172
Time × Low Arousal × Positive Valence	18	19.13	<.05	.545
Age × Time × Low Arousal × Positive Valence	18	17.10	<.05	.517
Time × Positive Valence × High Arousal	18	5.52	<.05	.256
Age × Time × Positive Valence × High Arousal	18	4.94	<.05	.236
Time × Negative Valence × Low Arousal	18	7.92	<.05	.331
Age × Time × Negative Valence × Low Arousal	18	6.85	<.05	.300
Time × Negative Valence × High Arousal	18	10.66	<.05	.400
Age × Time × Negative Valence × High Arousal	18	9.59	<.05	.375

**Table 5.** Prevalence of depression symptoms before waiting list, before and after the NF Intervention.

	t0	t1	t2
PHQ-9			
<10	12 (66.7%)	13 (72.2%)	12 (66.7%)
≥10	6 (33.3%)	5 (27.8%)	6 (33.3%)
N = 18			

*Note:* PHQ-9 = Patient Health Questionnaire-9, sum scores of ≥ 10 indicate major depression symptoms.

### Depression Prevalence

The prevalence of major depression symptoms (PHQ-9 sum scores ≥ 10) immediately after inclusion and before waiting period was 33.3% (t0) (Table 5). After a 5-week waiting period before intervention, it decreased to 27.8% (t1), and increased again to 33.3% (t2, after intervention).

### Depression State

The mean PHQ-9 after inclusion was 8.88 (4.581). Before intervention it was 7.71 (4.254) and decreased during intervention to 6.71 (4.165). The analysis of variance with repeated measures showed a significant effect with *F*(2,14) = 4.361, *p *< .05, *η^2^* = .384, *N = *17. The results of the Wilcoxon signed rank test comparing the depression state by PHQ-9 before waiting period and NF intervention (*Z *= -1.698, *p *= .089, *N *= 18) and before and after NF intervention (*Z *= -1.912, *p *= .056., *N *= 17) revealed no significant differences. Figure 4 shows the mean values of the depression state (PHQ-9) before waiting period (t0), before (t1) and after (t2) NF treatment.

A high degree of internal consistency was found between t0, t1, and t2 PHQ-9 measurements with Cronbach's Alpha value *α* = .904 and average measure ICC of .904 with a 95% confidence interval from .790 to .961 (*F*(17,34) = 10.461, *p *< .001).

The Pearson correlation coefficient showed a significant relationship between t1 and t2 measurements (*r*(*16*) = .803, *p* = < .001).

### Effect of age on Depression State

The ANOVA with repeated measures using age (t0) as the covariate showed a significant main effect (*F*(2,14) = 5.533, *p *< .05, *η^2^* = .269, *N *= 17) and a trend of the age interaction (*F*(2,14) = 3.484, *p *= .053, *η^2^* = .189, *N *= 17). During the 5-week waiting period, patients <55 years of age showed a decrease in mean PHQ-9 score of 1.44 (3.358) points, while those ≥55 had a reduction of 0.71 (2.059) (Figure 5). With treatment, the younger patients achieved a mean relief of 1.67 (2.291) points, while the older patients showed no mean change in PHQ-9 scores 0.00 (0.816). Post-hoc tests were calculated with Bonferroni correction showing significant differences between measurement points t0 and t2 (*p *= .016). No significance for t1 compared with t0 (p = .259) or t2 (*p *= .666) was found.

Depressive state was not correlated with age (*rho *= -.244, *p *= .346 for t0; *rho *= -.095, *p *= .716 for t1; *rho *= .212, *p *= .414).

## Discussion

To the best of our knowledge, this is the first investigation of the effects of an NF intervention on depression in cancer patients and survivors and its association with emotion processing using the P300.

Our data showed a significant reduction in P300 amplitude from pre to post therapy. Considering individual conditions, the following emerged: In the high arousal level, P300 amplitudes triggered by negative valence stimuli were larger than the amplitudes triggered by positive valence stimuli before therapy. During the course of therapy, the negative valence stimuli tended to show a greater decrease than the positive ones, so that both decreased to a similar level after therapy.

In the positive valence dimension, the amplitudes triggered by low arousal stimuli tended to decrease more than those triggered by high arousal stimuli during the course of NF training.

Concerning depression, we observed a markedly high prevalence in our patient cohort. Mean depression symptomatology as measured by the PHQ-9 showed a decrease from 8.88 before waiting list to 7.71 before the intervention to 6.71 after the intervention, thus showing a reduction approximately 2 points on the Likert scale from t0- t2. An age effect was observed, showing that patients younger than 55 years in particular had a greater reduction in depressive symptomatology.

Previous investigations on ERP changes in depressive symptoms showed a reduction in the P300 amplitude compared to healthy individuals^13–15^ and an increase after symptom improvement.^19,20^ According to these studies, we would have expected that, as a correlate to the decreasing burden of depression in our cohort, P300 amplitudes would have tended to increase. The current findings are not in line with previous research, which might be due to the fact that the present cohort does not consist of patients with major depression but cancer patients and survivors with depressive symptoms. In addition, our subjects received an intervention targeting brainwave activity, which to the best of our knowledge had not been previously investigated in any study. Moreover, P300 amplitudes might naturally decrease over time of a study due to habituation effects to the stimuli. Therefore, the results of this study have to be interpreted with caution. Furthermore, future studies should include analyses of P300 amplitudes over all measurement time points (t0, t1 and t2), which was not possible in this study due to economic reasons.

Our pre therapy observation that among high arousal stimuli, negative pictures triggered higher P300 amplitudes than positive pictures is consistent with earlier studies.^12,47–49^ Most of these studies were conducted as a single-stage oddball experiment rather than a pre-post investigation in the context of an intervention. Ito et al described^48^ the overall stronger reactions to negative stimuli as negativity bias, meaning that negative stimuli influence cognitive assessments stronger than positive stimuli.

As Prinsloo et al showed,^50^ NF is an intervention with measurable brain outcomes and can achieve a reduction in peripheral neuropathy in cancer patients and survivors by increasing alpha activity and reducing beta activity.

In this context, the greater decrease of amplitudes generated by intensely negative pictures could be interpreted as a correlate of a more relaxed brain activity level due to the EEG frequency band modulation by NF (resulting in a more relaxed handling of pronounced negative stimuli).

Concerning the overall reduction of P300 amplitudes, the trend that amplitudes triggered by high arousal pictures do not decrease in the same amount as those triggered by low arousal pictures could emphasize the importance of strong positive stimuli.

The tendency to preserve the reaction to strong positive stimuli while showing more balanced reactions to mild positive and strong negative stimuli could be interpreted as a correlate of a generally more moderate state of mind due to the decrease of depression load.

The present study demonstrated a high prevalence of depression in cancer patients and survivors, which is in line with existing literature.^51–53^ A clinical need for intervention is seen by many authors (see^54–58^). To date, NF as a non-invasive but evidence-based alternative medical method is rarely used in this patient cohort^26^ although it is already well established and well-researched in depressive disorders.^27,35,36,59–62^ These studies also suggest an alleviation of depressive symptoms by NF in the cohort of cancer patients and survivors. In our findings, depressive symptoms were not correlated with the age of the patients. However, a larger intervention effect for patients younger than 55 years of age was detected. These results suggest that younger patients would potentially benefit more from an NF intervention. Since there are only few efficacy studies for NF therapy in the field of psycho-oncology,^26^ this could be an important implication for future therapy offers in this patient group.

### Limitations

Clearly, many statistical analyses did not reach the significance level due to the small sample size of 18 participants. In the context of the relatively small number of subjects, the lack of a cross-over design is also a weak point. Moreover, the conduct of the study was hampered by the shutdown during the COVID-19 pandemic. We believe that a study with a larger sample would confirm the statistical trends and show more significant results. Furthermore, it is limiting that this study was only designed as an observational trial. In future surveys, the change in P300 amplitude from t0 to t1 (5 weeks without NF intervention) in addition to psychometrics should be used as a control condition. This was not possible at the time of our survey for this sub-study due to economic reasons. Moreover, another limitation is that the P300 amplitude was measured at coordinate Pz. However, literature suggests a maximal amplitude at right medial coordinates,^63,64^ which should be taken into account in future studies.

## Conclusion

In conclusion, the decrease of the P300, especially to intensely negative pictures, could reflect a therapeutic effect on brain activity. Further studies are necessary to investigate the profound correlation between brain wave modification by NF therapy and event-related potentials.

Moreover, this study provides initial evidence that EEG-NF may alleviate depressive symptoms in cancer patients and cancer survivors. Especially patients under 55 years of age may benefit from this therapy. However, this should be validated by future studies with larger sample sizes.
